# A cross-sectional study of the development of volitional control of spatial attention in children with chromosome 22q11.2 deletion syndrome

**DOI:** 10.1186/1866-1955-4-5

**Published:** 2012-02-15

**Authors:** Heather M Shapiro, Yukari Takarae, Danielle J Harvey, Margarita H Cabaral, Tony J Simon

**Affiliations:** 1 MIND Institute and Department of Psychiatry and Behavioral Sciences, University of California, Davis, 2825 50th Street, Sacramento, CA 95817, USA; 2 Center for Mind and Brain, 267 Cousteau Place, University of California, Davis, Davis, CA 95618, USA; 3 Division of Biostatistics, Department of Public Health Sciences, School of Medicine, One Shields Avenue, Med Sci 1-C, University of California, Davis, Davis, CA 95616, USA

**Keywords:** 22q11.2 deletion syndrome, Velo-cardio-facial syndrome, spatial attention, childhood cognitive development, developmental disorders

## Abstract

**Background:**

Chromosome 22q11.2 deletion syndrome (22q11.2DS) results from a 1.5- to 3-megabase deletion on the long arm of chromosome 22 and occurs in approximately 1 in 4000 live births. Previous studies indicate that children with 22q11.2DS are impaired on tasks involving spatial attention. However, the degree to which these impairments are due to volitionally generated (endogenous) or reflexive (exogenous) orienting of attention is unclear. Additionally, the efficacy of these component attention processes throughout child development in 22q11.2DS has yet to be examined.

**Methods:**

Here we compared the performance of a wide age range (7 to 14 years) of children with 22q11.2DS to typically developing (TD) children on a comprehensive visual cueing paradigm to dissociate the contributions of endogenous and exogenous attentional impairments. Paired and two-sample t-tests were used to compare outcome measures within a group or between groups. Additionally, repeated measures regression models were fit to the data in order to examine effects of age on performance.

**Results:**

We found that children with 22q11.2DS were impaired on a cueing task with an endogenous cue, but not on the same task with an exogenous cue. Additionally, it was younger children exclusively who were impaired on endogenous cueing when compared to age-matched TD children. Older children with 22q11.2DS performed comparably to age-matched TD peers on the endogenous cueing task.

**Conclusions:**

These results suggest that endogenous but not exogenous orienting of attention is selectively impaired in children with 22q11.2DS. Additionally, the age effect on cueing in children with 22q11.2DS suggests a possible altered developmental trajectory of endogenous cueing.

## Background

Chromosome 22q11.2 deletion syndrome (22q11.2DS) results from a 1.5- to 3-megabase microdeletion on the long (q) arm of chromosome 22 [1] and occurs in approximately 1 in 4000 live births [2,3]. Children with this disorder have a physical phenotype that might include cardiovascular abnormalities and cleft palate [4], in addition to mild to moderate learning impairments and a characteristic cognitive phenotype [5,6]. More specifically, the characteristic cognitive profile in most children with 22q11.2DS includes non-verbal impairments that stand in contrast to relative strengths in the verbal domain [7]. These non-verbal impairments include, but are not limited to, impairments in attention, spatial cognition, quantitative cognition, and arithmetical processing [8-10].

Simon recently proposed that underlying impairments in attention may subserve many of the other cognitive impairments in children with 22q11.2DS [11]. In this view, the atypical development of attentional processes, mediated by the genetics of the disorder, cascades into impairments in magnitude and numerical processing. Subsequently, these impairments play a fundamental role in the characteristic impairments in non-verbal cognitive function that are recognized as a hallmark of 22q11.2DS. Thus, a better understanding of attentional development in children with 22q11.2DS is critical for identifying an endophenotype of the disorder that is potentially a root of their cognitive impairments and could be targeted early in development for therapeutic intervention.

Attention is an important cognitive process by which task relevant information is selected at the cost of diminished representations of distracting stimuli [12]. It regulates cognitive processes at many levels and sensory modalities, including a critical modulatory role of vision [13]. In the context of daily life, people are exposed to a broad range of visual stimuli, not all of which are relevant for their current goals or behavior. Visuospatial attention helps to focus on spatial locations in the environment that contain the most relevant visual stimuli. Subsequently, response time to a stimulus within the attended spatial location will be quicker than response time to a stimulus outside of that location [14]. This effect is believed to be due to amplified signal processing within the attended location [15].

Visuospatial attention is not a unitary process, but rather consists of two primary mechanisms by which one orients attention [16]. One mode involves voluntary or endogenous shifts of attention to a relevant location in space. The other mode of attentional orienting involves attention capture by a salient external stimulus; this is referred to as exogenous attention. Both endogenous and exogenous orienting are important for behavior. Endogenous orienting allows one to focus attention on a specific spatial location based on a particular goal or task at hand. Exogenous orienting, on the other hand, enables one to respond to the sudden appearance or onset of a stimulus that may provide critical new information. These two mechanisms are not mutually exclusive. A sudden exogenous stimulus might interrupt endogenous attention at a particular location, while endogenous attention might also be utilized to prevent continuous interruptions at the periphery. Within this context, visual attention can be best understood as a process of finite capacity that resolves competing inputs by either responding reflexively to changes in the environment, or by selecting goal-directed, volitional orientation [12,17].

Previous research suggests that children with 22q11.2DS might have impairments in visuospatial attention [11,18-20]. One study tested endogenous orienting in children with 22q11.2DS [8] using the classic Posner cueing task, which requires participants to respond to a stimulus that appears at lateral spatial locations following either a valid or invalid central symbolic spatial cue [14]. In the study by Simon *et al.*, children with 22q11.2DS demonstrated a significantly longer response time following an invalid endogenous, central arrow, cue when compared to typically developing children, suggesting impaired attention orienting when disengaging from an invalidly cued location and reorienting to the location at which a target appeared. Another recent study also examined visual spatial attention in children with 22q11.2DS [21] by measuring their "orienting index", which is a subcomponent of the attention network test that compares the effects of invalid relative to valid cues in an exogenously cued task design. In contrast to the previous study that examined endogenous orienting, the results of this study did not show a greater invalidity cost in children with 22q11.2DS, although their response times were significantly slower to all cue types than those of typically developing children. Collectively, these studies demonstrate different levels of impairment with respect to different attentional modes in children with 22q11.2DS, suggesting a dissociation between endogenous relative to exogenous orienting in this population. Unfortunately, however, it is difficult to critically evaluate these differences since they stem from separate studies that involved different participants performing different experiments. In order to draw more rigorous conclusions about the nature of attention orienting in children with 22q11.2DS, it is necessary to carry out an experiment that compares the performance of the same individuals on the same task relative to typically developing children. Thus, this is one of the aims of the current study.

The second aim of this study was to examine possible developmental differences in visuospatial attention in children with 22q11.2DS relative to typically developing (TD) children. It is important to consider that 22q11.2DS is a neurodevelopmental disorder and thus will have differential effects on mind and brain particularly during early and middle childhood. Furthermore, evidence suggests that endogenous and exogenous modes of orienting follow different developmental time courses [17]. Typically developing young children are capable of adult-like behavior in exogenous visual attentional cueing paradigms, suggesting that the underlying mechanisms of exogenous visual orienting develop early [22,23]. By contrast, endogenous orienting appears to follow a more protracted developmental time course. While children demonstrate the ability to orient their attention following an endogenous cue, it is not until approximately 8 years of age that they are able to sustain this volitional attention [24]. Developmental trajectories of attentional orienting have not been examined in 22q11.2DS, despite their likely functional significance. The developmental span of attentional dysfunction in 22q11.2DS is not known but even if it eventually resolves, compromised attentional functioning during childhood will affect many aspects of cognitive development, including academic achievement.

In sum, the overarching goals of the current study were to examine the nature and extent of impairments in endogenous and exogenous visuospatial attention through middle to late childhood development in 22q11.2DS. This was accomplished by testing children with 22q11.2DS and age-matched typically developing (TD) children on a behavioral task that presents endogenous and exogenous cueing conditions in a within-subjects design. In order to examine possible developmental differences, age effects were examined in a cross-sectional analysis across the age range of 7 to 14 years.

In the results of the current study we found that, as a group, 7 to 14-year-old children with 22q11.2DS showed impaired reorienting to invalidly cued locations under endogenous, but not exogenous, cueing task demands. Additionally, these impairments were due exclusively to performance differences in younger children with 22q11.2DS. Older children with 22q11.2DS did not demonstrate impairments on these tasks relative to TD children, and no age effects were found in the TD group. Thus, the results indicate a selective atypical developmental trajectory of at least one aspect of attentional control in children with 22q11.2DS. Specifically, younger children are impaired relative to TD individuals in endogenous but not exogenous orienting of attention conditions while older children with 22q11.2DS were not significantly impaired relative to their TD peers.

## Methods

### Participants

Forty-six children with chromosome 22q11.2 deletion syndrome (21 female, 25 male) and thirty-seven typically developing (TD) comparison children (18 female, 19 male), from 7 to 14 years of age, participated in the study. In order to examine age effects, each diagnostic group was divided into younger and older age groups by a median split. Thus, a younger group consisted of participants from 7.0 to 10.9 years of age, and an older group consisted of participants from 11.0 to 14.9 years of age. In the children with 22q11.2DS, the deletion was confirmed by fluorescence *in situ *hybridization (FISH) testing. Data on IQ (from WASI or WISC III/IV) were available for 44 children with 22q11.2DS and 33 TD participants. Range of full-scale IQ (FSIQ) was 52 to 103 for children with 22q11.2DS and 92 to 135 for TD children. Attention deficit/hyperactivity disorder (ADHD) was diagnosed in accordance with the parent-rated Swanson, Nolan, and Pelham IV Rating Scale (SNAP-IV) [25] and was assessed in 32 participants with 22q11.2DS. The parents of all participants provided written informed consent based on protocols approved by the Institutional Review Board at the University of California, Davis. Table 1 depicts the demographic information for children in each group.

**Table 1:** Demographic data on children with 22q11.2DS and TD children

		22q11.2DS		TD	
		***n***	**Mean age in years (SD)**	***n***	**Mean age in years (SD)**

Gender	Female	21	10.38 (1.96)	18	9.45 (2.32)
	Male	25	10.70 (2.42)	19	10.55 (2.14)
Relative age	Younger	28	9.09 (1.22)	25	8.69 (1.24)
	Older	18	12.83 (1.21)	12	12.77 (1.11)
Total		46	10.56 (2.20)	37	9.99 (2.29)

### Procedure

**Figure 1:**
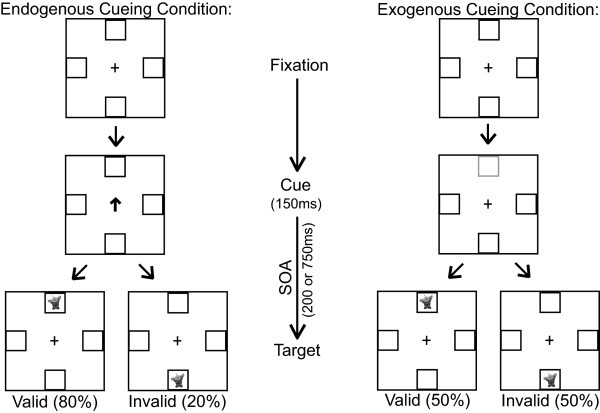
**Endogenous-exogenous cueing task**.

All participants completed the "endogenous-exogenous" cueing experiment (Figure 1). This experiment was adapted from Dennis *et al.*'s design (2005) where children are presented with four black squares that are located to the left, right, above, and below a central fixation cross [26]. The central fixation cross was 0.95° high and wide when viewed at a distance of 60 cm, and the squares were 1.91° in diameter, each located 3.82° from the center of the fixation cross. The participants were instructed to maintain fixation on the black cross and press a button when a target appeared in one of the boxes. Prior to the appearance of the target, however, the participants were presented with a cue for 150 ms. In the endogenous condition, the cue was a predictive central arrow that pointed to the likely location of the target. This arrow was valid (pointed to the correct location of the subsequent target) on 80% of the trials and invalid (pointed towards the opposite spatial location) on the other 20%. Thus, on any given trial, the target would only appear in one of two locations: the correct location or the one box located in the opposing spatial location on the other side of the fixation cross. In the exogenous cueing condition, the cue was a non-predictive luminance change in one of the peripheral boxes. This cue appeared with 50% invalid probability to avoid creating expectancy effects that could result in endogenous cueing. The time lapse from the offset of the cue to the onset of the target (also known as stimulus onset asynchrony or SOA) was either 200 ms or 750 ms in order to examine possible differences in the time course of cueing. Subsequently, the target was presented and participants responded via button press on a response pad that had buttons in spatial locations that mirrored those of the four boxes on the computer screen. Participants were instructed to press the button corresponding to the spatial location of the target.

After being given instructions and two demonstration trials (one for the endogenous and exogenous condition, respectively), the participants completed 10 practice trials of each. The full experiment contained 80 trials of each cueing condition, broken into two blocks of 40 trials for each condition. The blocks were interleaved so that an endogenous block followed an exogenous block and vice versa.

### Data analysis

In order to examine overall performance differences between groups, we measured participants' response time and accuracy for each combination of cueing conditions. Response time (RT) was measured as the time lapse from the onset of the target alien to the participant's button press. Only trials in which participants responded correctly were selected for analysis, and trials with response times less than 150 ms were excluded as anticipatory. Additionally, trials outside of 2.5 standard deviations from their mean of a particular condition were excluded as outliers. The median RT for each condition was selected from the remaining trials.

Since children with developmental disorders typically have lower accuracy on behavioral tasks, we combined RT with accuracy to generate an adjusted response time that takes performance into account. We calculated adjusted RT by dividing the raw RT by the accuracy for a particular condition using the formula *adjusted RT *= RT/accuracy. Using this adjustment, the RT remains unchanged with 100% accuracy and is increased in proportion to the number of errors. This measure accounts for speed/accuracy trade-offs and has been used to examine spatial cueing in children in previous studies [8,19,27]. Paired t-tests and two-sample t-tests were used to compare adjusted median RTs between conditions within a group or between groups.

Ultimately, the adjusted median RT was used to generate cue cost, a comprehensive outcome measure that describes the cost of reorienting attention following an invalid relative to a valid cue. We calculated cue cost by subtracting the adjusted median valid RT from the adjusted median invalid RT. A larger cue cost is indicative of a longer time necessary to disengage from an invalidly cued location, and can be interpreted as impaired attention orienting.

Due to the design of the task, there are multiple measurements per person. These include cue type: endogenous, exogenous; direction: right, left, up, down; and SOA: 200, 750 ms. In order to utilize all of the data available for each individual and to test specific hypotheses regarding differences between TD children and children with 22q11.2DS, repeated measures regression models were fit to the data. Initial models included main effects for cue type, diagnosis, target direction, SOA, and age (dichotomized as young/old by median split) as well as the interaction between diagnosis and cue-type to directly test for differences between the groups on the types of cues. Further models investigated differences by age including interactions between age and cue type, age and diagnosis, and the three-way interaction between age, diagnosis, and cue type. An interaction between SOA and diagnosis was also considered to assess differences between groups on SOA. An exchangeable correlation structure was assumed for the repeated measures allowing for different variability between the groups, because performance of children with 22q11.2DS is much more variable than that of TD children. Assumptions of this model were checked and were met by the data. All analyses were conducted in SAS using a confidence level of 0.95 (alpha = 0.05).

## Results

### General cueing effects

**Figure 2:**
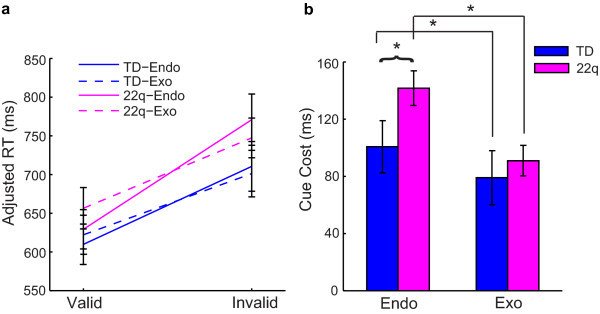
**General results of the endogenous-exogenous cueing task**.

Consistent with existing data, both groups had significantly greater response times to invalid relative to valid trials for endogenous and exogenous cueing conditions (*P *< 0.001 for both groups, both conditions) (Figure 2a). Additionally, the cue cost (response time difference between invalid minus valid trials) was significantly larger for all children on endogenous relative to exogenous trials for both groups (*P *< 0.001) (Figure 2b). Finally, children with 22q11.2DS had significantly larger cue cost when compared to TD children on endogenous (*P *< 0.001), but not exogenous trials (*P *= 0.10). Although children with 22q11.2DS appear to respond more slowly to all trial types (valid and invalid) relative to TD children, these differences were not statistically significant (*P *> 0.1) (Figure 2a).

### Cueing and age effects

**Figure 3:**
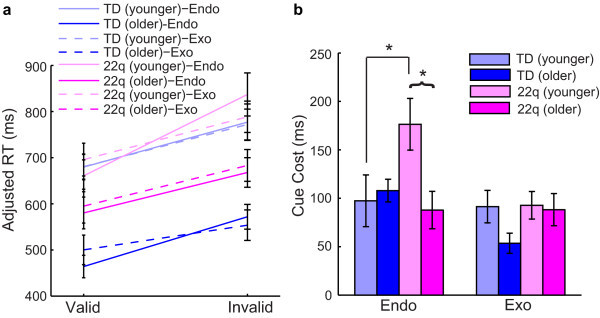
**Age results of the endogenous-exogenous cueing task**.

For deeper analysis, we divided the two groups into younger (7.0 to 10.9 years) and older (11.0 to 14.9 years) age groups by a median split. Overall, older TD children had significantly quicker response times on endogenous and exogenous trials relative to their younger counterparts (*P *< 0.001 for valid and invalid trials for both cueing conditions) (Figure 3a). Older children with 22q11.2DS were only significantly faster than their younger counterparts on invalid endogenous trials (*P *= 0.005). Younger children with 22q11.2DS had significantly larger cue cost on endogenous trials when compared to older children with 22q11.2DS (*P *< 0.001) (Figure 3b). In contrast, there was no difference in performance by age for TD children on endogenous trials (*P *= 0.99). Older children with 22q11.2DS performed comparably to older TD children (*P *= 0.54) as well as to younger TD children (*P *= 0.47) on endogenous trials. Thus, the group difference in performance on endogenous trials is due exclusively to performance differences in younger children with 22q11.2DS. Alternative models using age in years found that an increase of one year of age was associated with greater improvement in children with 22q11.2DS than TD children on endogenous trials (*P *= 0.004) supporting the results found using a median split of the ages. In particular, an increase of one year of age in children with 22q11.2DS was associated with a 34.5-unit decrease in cue cost on endogenous trials, while in TD children the decrease was only 2.5 units. In contrast to endogenous cueing, children with 22q11.2DS did not show a change in cue cost as a function of age on exogenous trials (*P *= 0.35) (Figure 3b). The same was true with TD children (*P *= 0.19). Therefore, these findings further strengthen the evidence for a different pattern of endogenous but not exogenous orienting in children with 22q11.2DS compared to their age-matched typical counterparts.

### Effects of cue location

**Figure 4:**
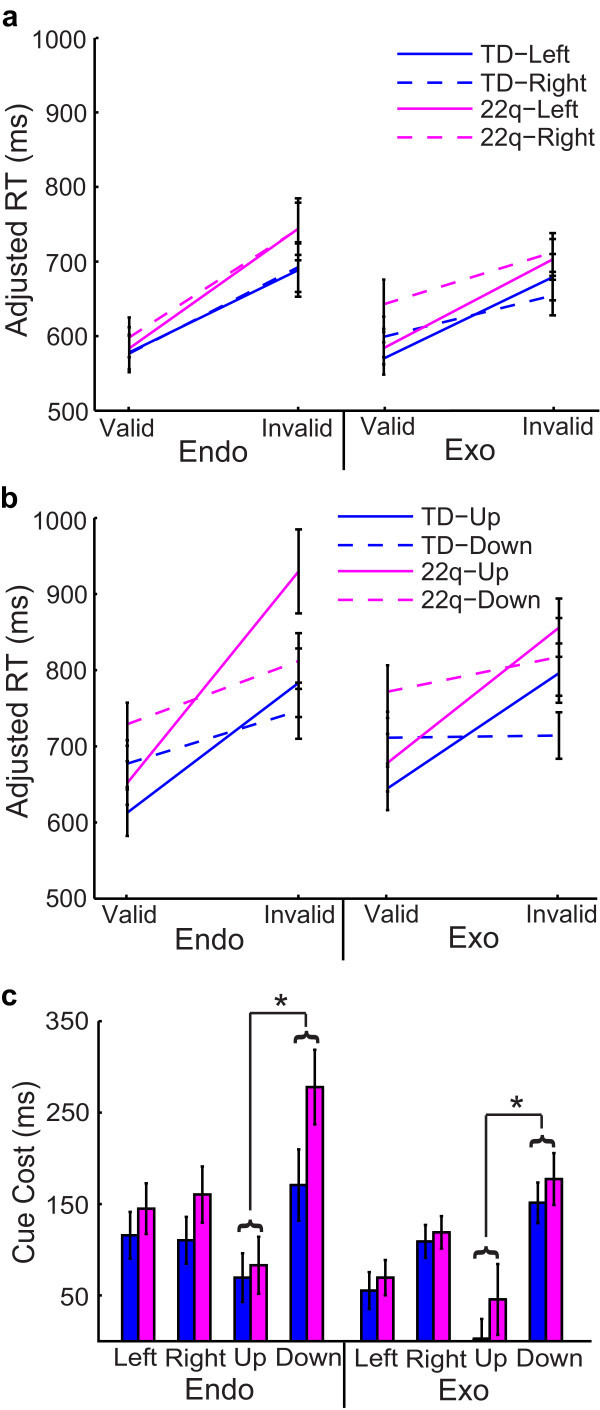
**Spatial location effects on the endogenous-exogenous cueing task**.

We next looked at response time to valid and invalid endogenous cues as a function of the target's final location (left, right, up, or down, respectively). We directly compared these response times for targets on the horizontal meridian (appearing to the left or right of fixation) (Figure 4a) versus the vertical meridian (targets appearing up or down relative to fixation) (Figure 4b). On both endogenous and exogenous trials, children with 22q11.2DS and TD children had significantly smaller cue cost for trials that were cued in the upward location relative to downward cues (*P *< 0.001) (Figure 4c).

On endogenous trials, younger children with 22q11.2DS produced larger cue costs when compared to older children with 22q11.2DS for all four cue locations (*P *< 0.001). By contrast, TD children did not show any difference in performance as a function of age on endogenous trials (*P *= 0.99). Young children with 22q11.2DS produced larger cue cost for spatial cues in the downward location when compared to young TD children (*P *< 0.001), but this difference was not true for older children (*P *= 0.54). On exogenous trials, there were no significant differences between younger and older children on cue location for either TD children (*P *= 0.19) or children with 22q11.2DS (*P *= 0.35). Younger children with 22q11.2DS were similar to younger TD children (*P *= 0.24) for all spatial cue locations on exogenous trials. Older children with 22q11.2DS also performed comparably to TD children for all cue locations on exogenous trials (*P *= 0.27).

### Effects of SOA

**Figure 5:**
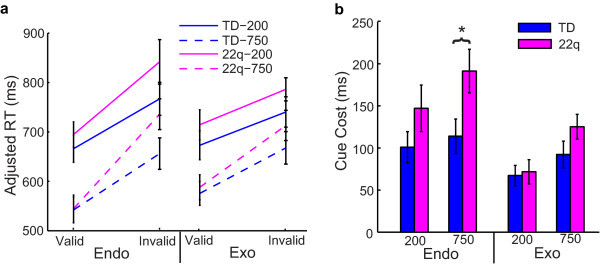
**SOA effects on the endogenous-exogenous cueing task**.

On both endogenous and exogenous trials, children with 22q11.2DS and TD children had faster response times to targets that appeared at 750 ms SOA relative to 200 ms (*P *< 0.005 for both groups, all conditions) (Figure 5a). On endogenous trials, children with 22q11.2DS had larger cue cost relative to TD children at an SOA of 750 ms (*P *= 0.02), but not at 200 ms (*P *= 0.17) (Figure 5b). Additionally, younger children with 22q11.2DS produced significantly larger cue costs relative to older children with 22q11.2DS at both 200 and 750 ms SOA on endogenous trials (*P *< 0.001). There was no difference in performance as a function of age for TD children at either SOA on endogenous trials (*P *= 0.99). Also on endogenous trials, young children with 22q11.2DS produced larger cue cost relative to young TD children at an SOA of 750 ms (*P *< 0.001) and 200 ms (*P *= 0.001). There were no differences in cue cost on endogenous trials between the groups by SOA at the older ages (*P *> 0.35). There were no group or age-related differences by SOA on cue cost for exogenous trials (*P *> 0.10) (Figure 5b).

### Clinical correlates

Although children with 22q11.2DS had significantly lower full-scale IQ (FSIQ) relative to TD children (*P *< 0.001), there was no correlation between FSIQ and endogenous or exogenous cue cost either for children with 22q11.2DS (*P *= 0.57 and *P *= 0.73) or TD children (*P *= 0.60 and *P *= 0.70). Of the 32 children with 22q11.2DS on whom we had information about ADHD status, 15 (47%) met DSM-IV criteria for ADHD. Comparing cue cost for individuals with ADHD relative to those without showed that there were no differences (*P *= 0.97 and *P *= 0.20 for endogenous and exogenous trials, respectively). For younger children with 22q11.2DS, 12 of 24 (50%) were diagnosed with ADHD relative to 3 of 8 (37.5%) older children with 22q11.2DS. There was no interaction of ADHD status and age either with cue cost for endogenous (*P *= 0.77) or exogenous (*P *= 0.53) trials.

## Discussion

This study replicates the facilitation effect seen in a Posner cueing paradigm, such that participants' response time (RT) to a cued location is quicker than to an uncued location [14]. It further replicates our previous finding that children with 22q11.2DS were impaired on an endogenous attentional task with central, symbolic cues [8]. Thus, the current study indicates that responses to invalid attentional cues are not globally impaired in children with 22q11.2DS but rather are selectively impaired under volitional but not reactive control and that this impairment might reduce with age. Although not statistically significant, it appeared that children with 22q11.2DS might respond more slowly to all trial types (valid and invalid) relative to TD children overall. This finding would be consistent with an effect seen on every other attentionally demanding task on which we have tested children with 22q11.2DS. We have consistently found that, on any task that requires the engagement of attention, children with 22q11.2DS demonstrate significantly slower RTs relative to their TD peers [28]. Thus, an overall group difference in RT would likely be a general attention effect that is not specific to the cueing task at hand. By looking at the difference scores (invalid minus valid RT), we will be able to best examine the specific group effects of endogenous and exogenous orienting.

Group differences in response to cue type (endogenous versus exogenous) revealed that children with 22q11.2DS had larger cue costs on endogenous trials relative to exogenous trials when compared to TD children. This suggests that our sample of children with 22q11.2DS have specific impairments in endogenous orienting when compared to age-matched TD individuals. On the other hand, children with 22q11.2DS performed comparably to TD children on exogenous trials, suggesting that they were not impaired when reflexively orienting to exogenous stimuli. After further analyzing the data, however, it became evident that the specific impairment in endogenous cueing might not be true of all children with 22q11.2DS.

An analysis of age effects revealed that the younger (7 to 10 years), but not older (11 to 14 years), children with 22q11.2DS were impaired on endogenous orienting of attention. This suggests that the orienting impairment seen in 22q11.2DS does not necessarily persist at the same level throughout childhood, but that initially delayed volitional orienting improves with age. This hypothesis will have to be directly tested in the future through longitudinal studies that are more appropriate for directly testing patterns of development. Evidence shows that, even in TD individuals, endogenous orienting of attention follows a more protracted time course of development relative to exogenous orienting, with endogenous orienting improving up to approximately 8 years of age [17]. Our results support that endogenous orienting was already fully developed in the younger TD individuals (ages 7 to 10 years), because there was no difference in performance between this group and the older TD individuals (ages 11 to 14 years). In contrast, the younger children with 22q11.2DS performed significantly less well than their older counterparts, suggesting that endogenous attentional control might be developmentally delayed in children with 22q11.2DS.

We also examined the effect of other variables in the endogenous-exogenous cueing task to see which factors might play a role in the observed impairment in endogenous cueing in younger children with 22q11.2DS. These variables included the different target locations (left, right, up, down) and SOAs of different lengths (200 ms and 750 ms, respectively). The motivation behind including these different factors was to examine possible differences in spatial and temporal elements of cueing. We found that, although there were no factors specific to endogenous cueing impairments in children with 22q11.2DS, there were interesting spatial and temporal effects that appeared collectively in both groups. First of all, both children with 22q11.2DS and TD children had significantly larger cue cost for cues toward the lower quadrant relative to the upper quadrant of the screen (Figure 4e). This difference was due to quicker response time to valid cues and slower response time to invalid cues in the downward direction relative to the upward direction (Figure 4c). The faster response time to valid cues in the downward direction is consistent with Previc's theory of visual search (1990), which posits that visual attention can be divided into two categories: peripersonal and extrapersonal. The peripersonal system pertains to activities requiring visual search within a range near to our bodies, for tasks that might involve reaching and other visuomotor coordination, and this system tends to be biased towards the lower visual field. On the other hand, the extrapersonal system is generally used in visual search and has bias towards the upper visual field. Since the endogenous-exogenous cueing task is a visuomotor task that takes place only 60 cm from the participant, then it would be the peripersonal attention system that mediates this task and it is here where children with 22q11.2DS appear to have a delayed developmental ability to volitionally control their attention. This suggests that children with 22q11.2DS have impaired attentional control within their peripersonal space, and this impairment plays a role in their overall neurocognitive phenotype. As Ladavas *et al.*'s review [29] demonstrates, even typical and brain-damaged adults demonstrate considerable activity-dependent plasticity in their non-unitary, body part-centered, representations of peripersonal space. Thus it is quite likely that, due to impairments in volitional control of endogenous attention, children with 22q11.2DS develop with atypical mappings of peripersonal space that affects their ability to accurately represent the relationship of their body parts to objects in space, of the relationship of objects in space to one another, and of the scale and shape of the space surrounding them. This might be a factor in the motor impairments that have been reported in young children with 22q11.2DS [30,31] as well as their developing representations of magnitude and number, which also seem to be scaffolded by peripersonal spatial representations implemented by parietal systems [32,33].

In addition to cueing visual attention at different spatial locations, we also included two different SOAs (200 ms and 750 ms, respectively) in order to examine the time course of attention orienting in children with 22q11.2DS and TD children. We found that both groups collectively had significantly longer response times to targets that appeared at an SOA of 200 ms relative to 750 ms (Figure 5a), a phenomenon that is characteristic for short versus long SOAs. Additionally, children with 22q11.2DS had a significantly larger cue cost relative to TD children only at an SOA of 750 ms (Figure 5b). We hoped that by comparing the effects of a short (200 ms) versus a long (750 ms) SOA, we might observe inhibition of return (IOR) in the two groups. IOR is a well-documented phenomenon that describes exogenous attention orienting as it follows a characteristic time course of early facilitation followed by later inhibition [34,35]. Within our data, we see that response times to targets appearing 750 ms after valid exogenous cues are greater than those appearing 750 ms after valid endogenous cues (Figure 5a). On the other hand, neither group showed a difference in response times for valid exogenous relative to valid endogenous cues at 200 ms SOA. The increase in response time following valid exogenous cues at 750 ms SOA is consistent with the inhibition of return phenomenon that occurs at longer SOAs. This difference was apparent in both groups and did not differ between groups.

After taking spatial and temporal factors into account, the most reliable factor that predicted impairment in endogenous cueing in children with 22q11.2DS was age. Younger children with 22q11.2DS were consistently impaired on endogenous trials when compared to both older children with 22q11.2DS and younger TD children. The impairment in endogenous cueing for younger children with 22q11.2DS might be related to abnormal brain structure or function. Structural imaging studies of children with 22q11.2DS demonstrated reductions in occipital, parietal, temporal, and cerebellar regions when compared to TD children [36-39]. Among the areas with greatest reductions in gray matter volume included regions of the posterior parietal lobes and posterior frontal cortex. Evidence also shows regions of enlargement in superior frontal cortex, the right insula, and superior, middle, and transverse temporal cortex [36]. In addition to gray matter differences, Simon *et al. *also reported differences in fractional anisotropy (FA), which characterizes the degree of coherence of water diffusion, and can be used as a measure of white matter integrity [40]. Specifically, they found that children with 22q11.2DS had abnormal patterns of FA relative to TD children in regions of the frontal and parietal lobes that are strongly associated with attentional function, and that these differences correlated differently in the two groups with performance on an endogenous cueing task. Barnea-Goraly *et al. *also found abnormal FA values in the left supramarginal and angular gyri in children with 22q11.2DS [41]. Most recently, Srivastava *et al. *reported age differences in the degree of cortical gyrification between children with 22q11.2DS and TD children within the age range of 6 to 15 years [42]. Importantly, the regions of cortex that significantly differed as a function of age included parietal structures that are functionally relevant, in typical individuals, for attentional tasks. Although a direct link is not explicitly clear, it is possible that these structural abnormalities might be related to the impairments in endogenous cueing in 22q11.2DS, given that endogenous cueing is largely mediated by a dorsal network that includes the dorsal posterior parietal cortex and superior frontal cortex [43].

Studies in adults with 22q11.2DS suggest that impairments in attention are persistent throughout adulthood when compared to typical controls [44,45]. Additionally, adults with 22q11.2DS demonstrate neuroanatomical abnormalities in regions shown to be important for attention [46-48]. Thus, existing evidence does not necessarily suggest that general attentional processing will continue to improve in older individuals with 22q11.2DS. However, visual spatial attention consists of a number of different components, not all of which share similar developmental courses or neural substrates. Our study was limited to a highly focused assessment of two critical components of the input selection component of visual attention [28]. Van Amelsvoort *et al. *[44] assessed the continuous performance vigilance aspect of attention in adults while Chow *et al. *[45] primarily assessed cognitive control using the Stroop and Trails A/B tests. Given broad differences between those studies and ours in the type of attentional processing being assessed, along with the fact that we have not tested adults with 22q11.2DS on our endogenous-exogenous cueing paradigm, we cannot speak to what a third and more mature developmental time point might look like for this component of attention. What we can say is that within our sample of 7 to 14-year-old children with 22q11.2DS, the older children performed comparably to typically developing peers on an endogenous cueing task on which the younger children were impaired. Thus, between the youngest and oldest ages at which we tested different children with 22q11.2DS, our data indicate that some strategy or compensatory processing appears to become available that mitigates the impairment seen in the younger age group. This poses very interesting questions about the specific components of attention that might be of relative strength at certain developmental time periods in this population and points to the very clear need for longitudinal studies in order to determine if this proposed account is supported by following young children through the critical early to middle school age range.

It is well established that rates of ADHD are highly elevated in children with 22q11.2DS [49]. Within our sample, 47% of the children with 22q11.2DS who were tested for ADHD met criteria for the disorder in accordance with DSM-IV criteria. The presence of this disorder, however, had no relation to performance on either the endogenous or exogenous cueing task. Thus, we can say that preexisting general impairments in clinical measures of attentional function do not account for the results that we see, and that the underlying impairment in endogenous orienting is not necessarily driving increased rates of ADHD. One reason that we might not see a correlation here is due to the specificity of the attentional component that is being measured in the endogenous-exogenous cueing task. Importantly, full-scale IQ also did not co-vary with performance on the task either in children with 22q11.2DS or TD children, thus eliminating IQ as a factor that might impact results. Other factors that would be important to consider include psychiatric diagnosis and medication status. Unfortunately, we do not have this data on our current sample, but it is information that warrants careful consideration in the future.

In sum, the age effect on endogenous cueing in 22q11.2DS poses interesting questions regarding neurocognitive development in this population. One possibility is that younger children with 22q11.2DS initially have an immature or atypically organized dorsal attention network that ultimately becomes more mature and/or typical at an age that is slightly delayed relative to TD children. Another possibility is that, with age, children with 22q11.2DS find other compensatory mechanisms for efficient endogenous orienting. These are questions that can be examined in the future by longitudinal behavioral and imaging studies. Additionally, since attention is critical to cognition, this posits the question as to whether or not the developmental delay in volitional control of attention is just another measure of developmental delay in 22q11.2DS, or if this is a more critical basic impairment that actually drives global delays as measured by IQ and school performance by compromising the ability to select and focus on salient information. In other words, given that endogenous attention is developmentally delayed, might this play a causative role in the abnormal development of other neural and cognitive processes? While this is not a question that can be answered within the scope of the present study, it is definitely an important question for future research.

## Conclusions

A cross-sectional analysis is an important preliminary step for a comprehensive evaluation of the development of attention in children with 22q11.2DS and TD children. When compared to TD children, children with 22q11.2DS demonstrate selective impairments in endogenous, but not exogenous, cueing. This suggests a specific attentional impairment in 22q11.2DS that is related to volitional, but not reflexive, orienting of attention. The specificity of the impairment in endogenous cueing, in conjunction with the age effect in 22q11.2DS, is suggestive of specific neural and cognitive characteristics that develop differently in children with 22q11.2DS and which may have a critical impact on the development of spatial and numerical mental representations and possibly on wider cognitive function.

## Abbreviations

22q11.2DS: chromosome 22q11.2 deletion syndrome; TD: typically developing; SOA: stimulus onset asynchrony; RT: response time.

## Competing interests

The authors declare that they have no competing interests.

## Authors' contributions

HMS played a primary role in data analysis and interpretation, in addition to drafting the manuscript. YT participated in the design of the study as well as data interpretation. DJH had a critical role in generating the statistical models for data analysis. MHC acquired the majority of the data, in addition to supporting data interpretation. TJS conceived of the study, participated in its design and coordination, and supported data analysis and interpretation. All authors read and approved the final manuscript.

## References

[B1] Carlson C Sirotkin H Pandita R Goldberg R McKie J Wadey R Patanjali SR Weissman SM Anyane-Yeboa K Warburton D Scambler P Shprintzen R Kucherlapati R Morrow BE Molecular definition of 22q11 deletions in 151 velo-cardio-facial syndrome patients Am J Hum Genet 1997 61 620 629 10.1086/515508 9326327 PMC1715959

[B2] Tezenas Du Montcel S Mendizabai H Ayme S Levy A Philip N Prevalence of 22q11 microdeletion J Med Genet 1996 33 719 8863171 10.1136/jmg.33.8.719 PMC1050716

[B3] Oskarsdottir S Vujic M Fasth A Incidence and prevalence of the 22q11 deletion syndrome: a population-based study in Western Sweden Arch Dis Child 2004 89 148 151 10.1136/adc.2003.026880 14736631 PMC1719787

[B4] Yamagishi H The 22q11.2 deletion syndrome Keio J Med 2002 51 77 88 10.2302/kjm.51.77 12125909

[B5] Moss EM Batshaw ML Solot CB Gerdes M McDonald-McGinn DM Driscoll DA Emanuel BS Zackai EH Wang PP Psychoeducational profile of the 22q11.2 microdeletion: A complex pattern J Pediatr 1999 134 193 198 10.1016/S0022-3476(99)70415-4 9931529

[B6] Wang PP Woodin MF Kreps-Falk R Moss EM Research on behavioral phenotypes: velocardiofacial syndrome (deletion 22q11.2) Dev Med Child Neurol 2000 42 422 427 10.1017/S0012162200000785 10875531

[B7] Woodin M Wang PP Aleman D McDonald-McGinn D Zackai E Moss E Neuropsychological profile of children and adolescents with the 22q11.2 microdeletion Genet Med 2001 3 34 39 10.1097/00125817-200101000-00008 11339375

[B8] Simon TJ Bearden CE Mc-Ginn DM Zackai E Visuospatial and numerical cognitive deficits in children with chromosome 22q11.2 deletion syndrome Cortex 2005 41 145 155 10.1016/S0010-9452(08)70889-X 15714897 PMC4318636

[B9] De Smedt B Swillen A Devriendt K Fryns JP Verschaffel L Ghesquiere P Mathematical disabilities in children with velo-cardio-facial syndrome Neuropsychologia 2007 45 885 895 10.1016/j.neuropsychologia.2006.08.024 17049567

[B10] De Smedt B Swillen A Verschaffel L Ghesquiere P Mathematical learning disabilities in children with 22q11.2 deletion syndrome: a review Dev Disabil Res Rev 2009 15 4 10 10.1002/ddrr.44 19213009

[B11] Simon TJ A new account of the neurocognitive foundations of impairments in space, time and number processing in children with chromosome 22q11.2 deletion syndrome Dev Disabil Res Rev 2008 14 52 58 10.1002/ddrr.8 18612330 PMC2442464

[B12] Luck SJ Gold JM The construct of attention in schizophrenia Biol Psychiatry 2008 64 34 39 10.1016/j.biopsych.2008.02.014 18374901 PMC2562029

[B13] Kastner S Ungerleider LG Mechanisms of visual attention in the human cortex Annu Rev Neurosci 2000 23 315 341 10.1146/annurev.neuro.23.1.315 10845067

[B14] Posner MI Orienting of attention Q J Exp Psychol 1980 32 3 25 10.1080/00335558008248231 7367577

[B15] Funes MJ Lupianez J Milliken B The role of spatial attention and other processes on the magnitude and time course of cueing effects Cogn Process 2005 6 98 116 10.1007/s10339-004-0038-7 18219508

[B16] Jonides J Irwin DE Capturing attention Cognition 1981 10 145 150 10.1016/0010-0277(81)90038-X 7198529

[B17] Ristic J Kingstone A Rethinking attentional development: reflexive and volitional orienting in children and adults Dev Sci 2009 12 289 296 10.1111/j.1467-7687.2008.00756.x 19143801

[B18] Bearden CE Woodin MF Wang PP Moss E McDonald-McGinn D Zackai E Emannuel B Cannon TD The neurocognitive phenotype of the 22q11.2 deletion syndrome: selective deficit in visual-spatial memory J Clin Exp Neuropsychol 2001 23 447 464 10.1076/jcen.23.4.447.1228 11780945

[B19] Simon TJ Bish JP Bearden CE Ding L Ferrante S Nguyen V Gee JC McDonald-McGinn DM Zackai EH Emanuel BS A multilevel analysis of cognitive dysfunction and psychopathology associated with chromosome 22q11.2 deletion syndrome in children Dev Psychopathol 2005 17 753 784 16262991 10.1017/S0954579405050364 PMC1360281

[B20] Bish JP Chiodo R Mattei V Simon TJ Domain specific attentional impairments in children with chromosome 22q11.2 deletion syndrome Brain Cogn 2007 64 265 273 10.1016/j.bandc.2007.03.007 17499412 PMC2727671

[B21] Stoddard J Beckett L Simon TJ Atypical development of the executive attention network in children with chromosome 22q11.2 deletion syndrome J Neurodev Disord 2011 3 76 85 10.1007/s11689-010-9070-3 21475729 PMC3056994

[B22] MacPherson AC Klein RM Moore C Inhibition of return in children and adolescents J Exp Child Psychol 2003 85 337 351 10.1016/S0022-0965(03)00104-8 12906846

[B23] Harman C Posner MI Rothbart MK Thomas-Thrapp L Development of orienting to locations and objects in human infants Can J Exp Psychol 1994 48 301 318 8069287 10.1037/1196-1961.48.2.301

[B24] Brodeur DA Enns JT Covert visual orienting across the lifespan Can J Exp Psychol 1997 51 20 35 9206322 10.1037/1196-1961.51.1.20

[B25] Swanson J School-Based Assessments and Interventions for ADD Students 1992 Irvine, CA: KC Publishing

[B26] Dennis M Edelstein K Copeland K Frederick J Francis DJ Hetherington R Blaser SE Kramer LA Drake JM Brandt ME Fletcher JM Covert orienting to exogenous and endogenous cues in children with spina bifida Neuropsychologia 2005 43 976 987 10.1016/j.neuropsychologia.2004.08.012 15716168

[B27] Enns JT Akhtar N A developmental study of filtering in visual attention Child Dev 1989 60 1188 1199 10.2307/1130792 2805896

[B28] Simon TJ Luck SJ Attentional Impairments in Children with Chromosome 22q11.2 Deletion Syndrome Cognitive Neuroscience of Attention 2011 2 New York, NY: Guilford Press 441 453

[B29] Ladavas E Serino A Action-dependent plasticity in peripersonal space representations Cogn Neuropsychol 2008 25 1099 1113 10.1080/02643290802359113 18726788

[B30] Gerdes M Solot C Wang PP Moss E LaRossa D Randall P Goldmuntz E Clark BJ Driscoll DA Jawad A Emanuel BS McDonald-McGinn DM Batshaw ML Zackai EH Cognitive and behavior profile of preschool children with chromosome 22q11.2 deletion Am J Med Genet 1999 85 127 133 10.1002/(SICI)1096-8628(19990716)85:2<127::AID-AJMG6>3.0.CO;2-F 10406665

[B31] Swillen A Devriendt K Legius E Eyskens B Dumoulin M Gewillig M Fryns JP Intelligence and psychosocial adjustment in velocardiofacial syndrome: a study of 37 children and adolescents with VCFS J Med Genet 1997 34 453 458 10.1136/jmg.34.6.453 9192263 PMC1050966

[B32] Longo MR Lourenco SF Bisecting the mental number line in near and far space Brain Cogn 2010 72 362 367 10.1016/j.bandc.2009.10.016 19951825

[B33] Zorzi M Priftis K Meneghello F Marenzi R Umilta C The spatial representation of numerical and non-numerical sequences: evidence from neglect Neuropsychologia 2006 44 1061 1067 10.1016/j.neuropsychologia.2005.10.025 16356515

[B34] Posner MI Cohen Y Bouma HB Components of visual orienting Attention and Performance Vol X 1984 Hillsdale, NJ: Erlbaum 531 556

[B35] Klein RM Inhibition of return Trends Cogn Sci 2000 4 138 147 10.1016/S1364-6613(00)01452-2 10740278

[B36] Simon TJ Ding L Bish JP McDonald-McGinn DM Zackai EH Gee J Volumetric, connective, and morphologic changes in the brains of children with chromosome 22q11.2 deletion syndrome: an integrative study Neuroimage 2005 25 169 180 10.1016/j.neuroimage.2004.11.018 15734353

[B37] Eliez S Schmitt JE White CD Reiss AL Children and adolescents with velocardiofacial syndrome: a volumetric MRI study Am J Psychiatry 2000 157 409 415 10.1176/appi.ajp.157.3.409 10698817

[B38] Campbell LE Daly E Toal F Stevens A Azuma R Catani M Ng V van Amelsvoort T Chitnis X Cutter W Murphy DG Murphy KC Brain and behaviour in children with 22q11.2 deletion syndrome: a volumetric and voxel-based morphometry MRI study Brain 2006 129 1218 1228 10.1093/brain/awl066 16569671

[B39] Kates WR Burnette CP Jabs EW Rutberg J Murphy AM Grados M Geraghty M Kaufmann WE Pearlson GD Regional cortical white matter reductions in velocardiofacial syndrome: a volumetric MRI analysis Biol Psychiatry 2001 49 677 684 10.1016/S0006-3223(00)01002-7 11313035

[B40] Simon TJ Wu Z Avants B Zhang H Gee JC Stebbins GT Atypical cortical connectivity and visuospatial cognitive impairments are related in children with chromosome 22q11.2 deletion syndrome Behav Brain Funct 2008 4 25 10.1186/1744-9081-4-25 18559106 PMC2443161

[B41] Barnea-Goraly N Eliez S Menon V Bammer R Reiss AL Arithmetic ability and parietal alterations: a diffusion tensor imaging study in velocardiofacial syndrome Brain Res Cogn Brain Res 2005 25 735 740 10.1016/j.cogbrainres.2005.09.013 16260124

[B42] Srivastava S Buonocore MH Simon TJ Atypical developmental trajectory of functionally significant cortical areas in children with chromosome 22q11.2 deletion syndrome Hum Brain Mapp 2012 33 213 223 10.1002/hbm.21206 21416559 PMC3212617

[B43] Corbetta M Shulman GL Control of goal-directed and stimulus-driven attention in the brain Nat Rev Neurosci 2002 3 201 215 11994752 10.1038/nrn755

[B44] van Amelsvoort T Henry J Morris R Owen M Linszen D Murphy K Murphy D Cognitive deficits associated with schizophrenia in velo-cardio-facial syndrome Schizophr Res 2004 70 223 232 10.1016/j.schres.2003.10.004 15329299

[B45] Chow EW Watson M Young DA Bassett AS Neurocognitive profile in 22q11 deletion syndrome and schizophrenia Schizophr Res 2006 87 270 278 10.1016/j.schres.2006.04.007 16753283 PMC3127863

[B46] van Amelsvoort T Daly E Robertson D Suckling J Ng V Critchley H Owen MJ Henry J Murphy KC Murphy DG Structural brain abnormalities associated with deletion at chromosome 22q11: quantitative neuroimaging study of adults with velo-cardio-facial syndrome Br J Psychiatry 2001 178 412 419 10.1192/bjp.178.5.412 11331556

[B47] Chow EW Zipursky RB Mikulis DJ Bassett AS Structural brain abnormalities in patients with schizophrenia and 22q11 deletion syndrome Biol Psychiatry 2002 51 208 215 10.1016/S0006-3223(01)01246-X 11839363 PMC3295830

[B48] van Amelsvoort T Daly E Henry J Robertson D Ng V Owen M Murphy KC Murphy DG Brain anatomy in adults with velocardiofacial syndrome with and without schizophrenia: preliminary results of a structural magnetic resonance imaging study Arch Gen Psychiatry 2004 61 1085 1096 10.1001/archpsyc.61.11.1085 15520356

[B49] Gothelf D Presburger G Levy D Nahmani A Burg M Berant M Blieden LC Finkelstein Y Frisch A Apter A Weizman A Genetic, developmental, and physical factors associated with attention deficit hyperactivity disorder in patients with velocardiofacial syndrome Am J Med Genet B Neuropsychiatr Genet 2004 126B 116 121 10.1002/ajmg.b.20144 15048660

